# The evolution of popular music: USA 1960–2010

**DOI:** 10.1098/rsos.150081

**Published:** 2015-05-06

**Authors:** Matthias Mauch, Robert M. MacCallum, Mark Levy, Armand M. Leroi

**Affiliations:** 1 School of Electronic Engineering and Computer Science, Queen Mary University of London, London E1 4NS, UK; 2 Division of Life Sciences, Imperial College London, London SW7 2AZ, UK; 3 Last.fm, 5-11 Lavingdon Street, London SE1 0NZ, UK

**Keywords:** popular music, diversity, stylistic revolutions, cultural evolution

## Abstract

In modern societies, cultural change seems ceaseless. The flux of fashion is especially obvious for popular music. While much has been written about the origin and evolution of pop, most claims about its history are anecdotal rather than scientific in nature. To rectify this, we investigate the US Billboard Hot 100 between 1960 and 2010. Using music information retrieval and text-mining tools, we analyse the musical properties of approximately 17 000 recordings that appeared in the charts and demonstrate quantitative trends in their harmonic and timbral properties. We then use these properties to produce an audio-based classification of musical styles and study the evolution of musical diversity and disparity, testing, and rejecting, several classical theories of cultural change. Finally, we investigate whether pop musical evolution has been gradual or punctuated. We show that, although pop music has evolved continuously, it did so with particular rapidity during three stylistic ‘revolutions’ around 1964, 1983 and 1991. We conclude by discussing how our study points the way to a quantitative science of cultural change.

## 2. Introduction

The history of popular music has long been debated by philosophers, sociologists, journalists, bloggers and pop stars [1–7]. Their accounts, though rich in vivid musical lore and aesthetic judgements, lack what scientists want: rigorous tests of clear hypotheses based on quantitative data and statistics. Economics-minded social scientists studying the history of music have done better, but they are less interested in music than the means by which it is marketed [8–15]. The contrast with evolutionary biology—a historical science rich in quantitative data and models—is striking, the more so because cultural and organismic variety are both considered to be the result of modification-by-descent processes [16–19]. Indeed, linguists and archaeologists, studying the evolution of languages and material culture, commonly apply the same tools that evolutionary biologists do when studying the evolution of species [20–25].

Until recently, the single greatest impediment to a scientific account of musical history has been a want of data. That has changed with the emergence of large, digitized, collections of audio recordings, musical scores and lyrics. Quantitative studies of musical evolution have quickly followed [26–30]. Here, we use a corpus of digitized music to investigate the history of American popular music. Drawing inspiration from studies of organic and cultural evolution, we view the history of pop music as a ‘fossil record’ and ask the kinds of questions that a palaeontologist might: has the variety of popular music increased or decreased over time? Is evolutionary change in popular music continuous or discontinuous? And, if it is discontinuous, when did the discontinuities occur?

To delimit our sample, we focused on songs that appeared in the US Billboard Hot 100 between 1960 and 2010. We obtained 30-s-long segments of 17 094 songs covering 86% of the Hot 100, with a small bias towards missing songs in the earlier years. Because our aim is to investigate the evolution of popular taste, we did not attempt to obtain a representative sample of all the songs that were released in the USA in that period of time, but just those that were most commercially successful.

**Figure 1.**
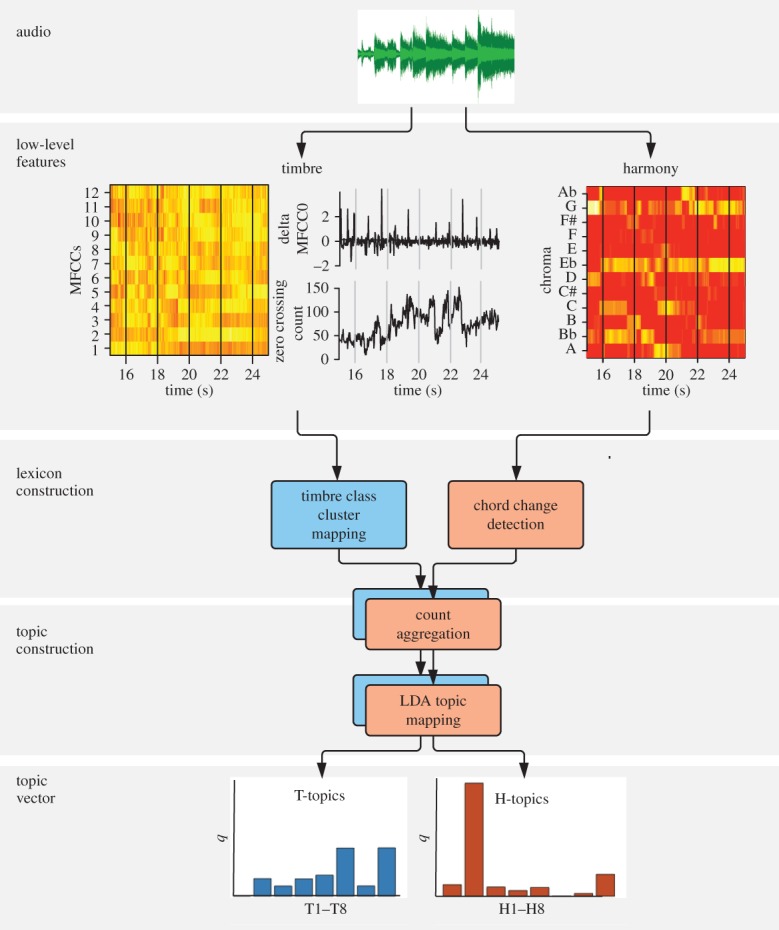
Data processing pipeline illustrated with a segment of Queen's *Bohemian Rhapsody*, 1975, one of the few Hot 100 hits to feature an astrophysicist on lead guitar.

Like previous studies of pop-music history [28,30], our study is based on features extracted from audio rather than from scores. However, where these early studies focused on technical aspects of audio such as loudness, vocabulary statistics and sequential complexity, we have attempted to identify musically meaningful features. To this end, we adopted an approach inspired by recent advances in text-mining (figure 1). We began by measuring our songs for a series of quantitative audio features, 12 descriptors of tonal content and 14 of timbre (electronic supplementary material, M2–3). These were then discretized into ‘words’ resulting in a harmonic lexicon (H-lexicon) of chord changes, and a timbral lexicon (T-lexicon) of timbre clusters (electronic supplementary material, M4). To relate the T-lexicon to semantic labels in plain English, we carried out expert annotations (electronic supplementary material, M5). The musical words from both lexica were then combined into 8+8=16 ‘topics’ using latent Dirichlet allocation (LDA). LDA is a hierarchical generative model of a text-like corpus, in which every document (here: song) is represented as a distribution over a number of topics, and every topic is represented as a distribution over all possible words (here: chord changes from the H-lexicon, and timbre clusters from the T-lexicon). We obtain the most likely model by means of probabilistic inference (electronic supplementary material, M6). Each song, then, is represented as a distribution over eight harmonic topics (H-topics) that capture classes of chord changes (e.g. ‘dominant-seventh chord changes') and eight timbral topics (T-topics) that capture particular timbres (e.g. ‘drums, aggressive, percussive’, ‘female voice, melodic, vocal’, derived from the expert annotations), with topic proportions *q*. These topic frequencies were the basis of our analyses.

## 3. Results

### 3.1 The evolution of topics

**Figure 2.**
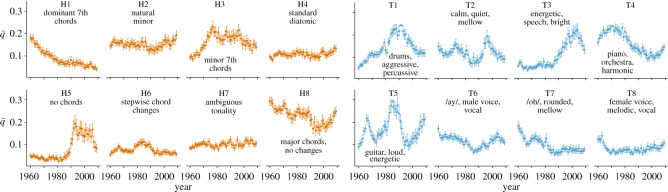
Evolution of musical topics in the Billboard Hot 100. Mean topic frequencies ($\bar{q}$) ±95% CI estimated by bootstrapping.

Between 1960 and 2010, the frequencies of the topics in the Hot 100 varied greatly: some topics became rarer, others became more common, yet others cycled (figure 2). To help us interpret these dynamics, we made use of associations between the topics and particular artists as well as genre-tags assigned by the listeners of Last.fm, a web-based music discovery service with approximately 50 million users (electronic supplementary material, M8). Considering the H-topics first, the most frequent was H8 (mean ± 95% CI: $\bar{q}=0.236\pm 0.003$)—major chords without changes. Nearly two-thirds of our songs show a substantial (>12.5%) frequency of this topic, particularly those tagged as classic country, classic rock and love (online tables). Its presence in the Hot 100 was quite constant, being the most common H-topic in 43 of 50 years.

Other H-topics were much more dynamic. Between 1960 and 2009, the mean frequency of H1 declined by about 75%. H1 captures the use of dominant-seventh chords. Inherently dissonant (because of the tritone interval between the third and the minor-seventh), these chords are commonly used in Jazz to create tensions that are eventually resolved to consonant chords; in Blues music, the dissonances are typically not resolved and thus add to the characteristic ‘dirty’ colour. Accordingly, we find that songs tagged blues or jazz have a high frequency of H1; it is especially common in the songs of Blues artists such as B.B. King and Jazz artists such as Nat ‘King’ Cole. The decline of this topic, then, represents the lingering death of Jazz and Blues in the Hot 100.

The remaining H-topics capture the evolution of other musical styles. H3, for example, embraces minor-seventh chords used for harmonic colour in funk, disco and soul—this topic is over-represented in funk and disco, and artists such as Chic and KC & The Sunshine Band. Between 1967 and 1977, the mean frequency of H3 more than doubles. H6 combines several chord changes that are a mainstay in modal rock tunes and therefore common in artists with big-stadium ambitions (e.g. Mötley Crüe, Van Halen, REO Speedwagon, Queen, Kiss and Alice Cooper). Its increase between 1978 and 1985, and subsequent decline in the early 1990s, marks the age of Arena Rock. Of all H-topics, H5 shows the most striking change in frequency. This topic, which captures the absence of identifiable chord structure, barely features in the 1960s and 1970s when, a few spoken-word-music collages aside (e.g. those of Dickie Goodman), nearly all songs had clearly identifiable chords. H5 starts to become more frequent in the late 1980s and then rises rapidly to a peak in 1993. This represents the rise of Hip Hop, Rap and related genres, as exemplified by the music of Busta Rhymes, Nas and Snoop Dog, who all use chords particularly rarely (online tables).

The frequencies of the timbral Topics, too, evolve over time. T3, described as ‘energetic, speech, bright’, shows the same dynamics as H5 and is also associated with the rise of Hip Hop-related genres. Several of the other timbral topics, however, appear to rise and fall repeatedly, suggesting recurring fashions in instrumentation. For example, the evolution of T4 (‘piano, orchestra, harmonic’) appears sinusoidal, suggesting a return in the 2000s to timbral qualities prominent in the 1970s. T5 (‘guitar, loud, energetic’) underwent two full cycles with peaks in 1966 and 1985, heading upward once more in 2009. The second, larger, peak coincides with a peak in H6, the chord changes also associated with stadium rock groups such as Mötley Crüe (online tables). Finally, T1 (‘drums, aggressive, percussive’) rises continuously until 1990, which coincides with the spread of new percussive technology such as drum machines and the gated reverb effect famously used by Phil Collins on *In the air tonight*, 1981. Accordingly, T1 is over-represented in songs tagged dance, disco and new wave and artists such as The Pet Shop Boys. After 1990, the frequency of T1 declines: the reign of the drum machine was over.

### 3.2 The varieties of music

**Figure 3.**
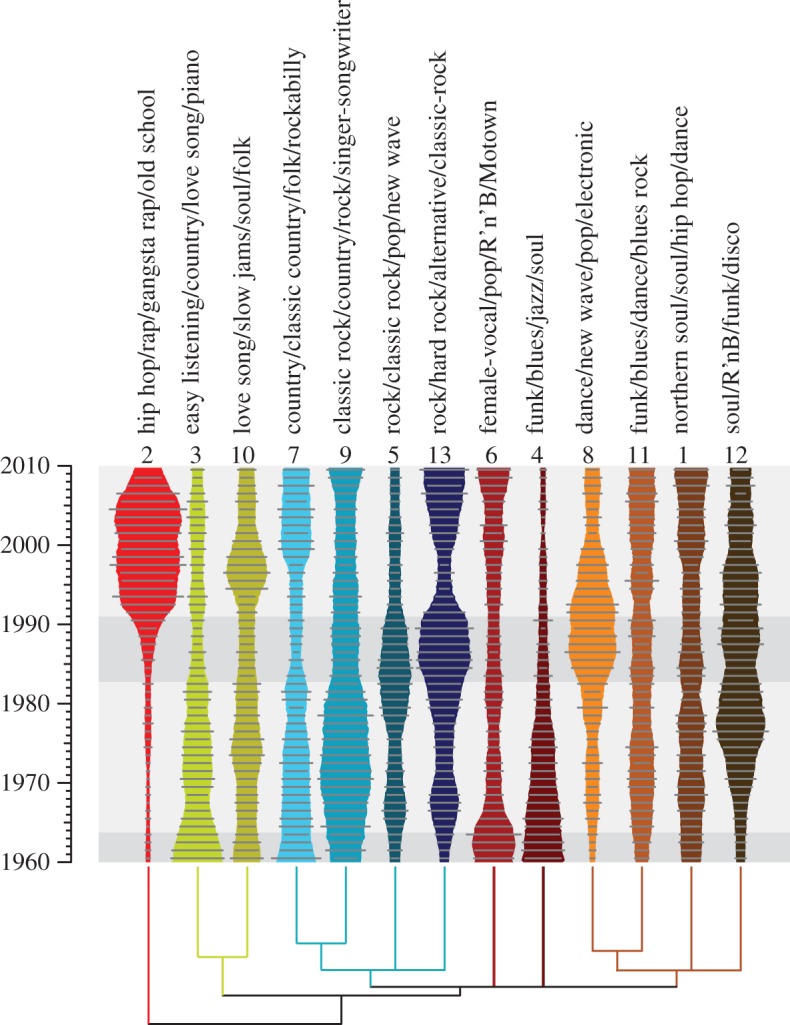
Evolution of musical styles in the Billboard Hot 100. The evolution of 13 styles, defined by *k*-means clustering on principal components of topic frequencies. The width of each spindle is proportional to the frequency of that style, normalized to each year. The spindle contours are based on a ±2-year moving average smoother; unsmoothed yearly frequencies are shown as grey horizontal lines. A hierarchical cluster analysis on the *k*-means centroids grouped our styles into several larger clusters here represented by a tree: an EASY-LISTENING + LOVE-SONG cluster, a COUNTRY + ROCK cluster and SOUL + FUNK + DANCE cluster; the fourth, most divergent, cluster only contains the HIP HOP + RAP-rich style 2. All resolved nodes have ≥75% bootstrap support. Labels list the four most highly over-represented Last.fm user tags in each style according to our enrichment analysis; see electronic supplementary material, table S1 for full results. Shaded regions define eras separated by musical revolutions (figure 5).

To analyse the evolution of musical variety, we began by classifying our songs. Popular music is classified into genres such as country, rock and roll, rhythm and blues (R`n'B) as well as a multitude of subgenres (dance-pop, synthpop, heartland rock, roots rock, etc.). Such genres are, however, but imperfect reflections of musical qualities. Popular music genres such as country and rap partially capture musical styles but, besides being informal, are also based on non-musical factors such as the age or ethnicity of performers (e.g. classic rock and K[orean]-Pop) [5]. For this reason, we constructed a taxonomy of 13 styles by *k*-means clustering on principal components derived from our topic frequencies (figure 3 and electronic supplementary material, M9). We investigated all *k*<25 and found that the best clustering solution, as determined by mean silhouette score, was *k*=13.

In order to relate Last.fm tags to the style clusters, we used a technique called enrichment analysis from bioinformatics. This technique is usually applied to arrive at biological interpretations of sets of genes, i.e. to find out what the ‘function’ of a set of genes is. Applying the GeneMerge enrichment-detection algorithm [31] to our style data, we found that all styles are strongly enriched for particular tags, i.e. for each style some Last.fm tags are significantly over-represented (electronic supplementary material, table S1), so we conclude that they capture at least some of the structure of popular music perceived by consumers. The evolutionary dynamics of our styles reflect well-known trends in popular music. For example, the frequency of style 4, strongly enriched for jazz, funk, soul and related tags, declines steadily from 1960 onwards. By contrast, styles 5 and 13, strongly enriched for rock-related tags, fluctuate in frequency, whereas style 2, enriched for rap-related tags, is very rare before the mid-1980s but then rapidly expands to become the single largest style for the next 30 years, contracting again in the late 2000s.

What do our styles represent? Figure 3 shows that styles and their evolution relate to discrete subgroups of the charts (genres), and hierarchical cluster analysis suggests that styles can be grouped into a higher hierarchy. However, we suppose that, unlike organisms of different biological species, all the songs in the charts comprise one large, highly structured, metapopulation of songs linked by a network of ancestor–descendant relationships arising from songwriters imitating their predecessors [32]. Styles and genres, then, represent populations of music that have evolved unique characters (topics), or combinations of characters, in partial geographical or cultural isolation, e.g. country in the Southern USA during the 1920s or rap in the South Bronx of the 1970s. These styles rise and fall in frequency over time in response to the changing tastes of songwriters, musicians and producers, who are in turn influenced by the audience.

### 3.3 Musical diversity has not declined

Just as palaeontologists have discussed the tempo and mode of evolutionary change in the fossil record [33], historians of music have discussed musical change and the processes that drive it. Some have argued that oligopoly in the media industries has caused a relentless decline in cultural diversity of new music [1,2], whereas others suggest that such homogenizing trends are periodically interrupted by small competitors offering novel and varied content resulting in ‘cycles of symbol production’ [8,12]. For want of data, there have been few tests of either theory [9–11,14].

**Figure 4.**
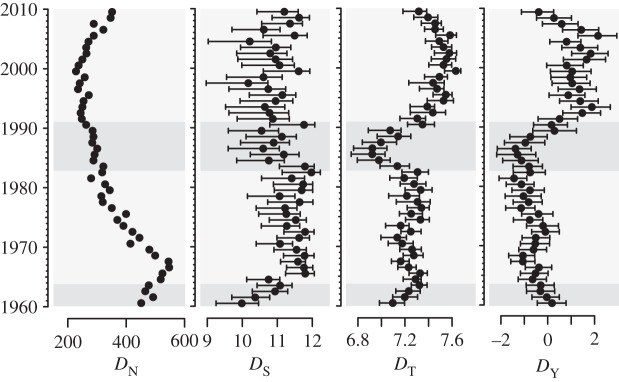
Evolution of musical diversity in the Billboard Hot 100. We estimate four measures of diversity. From left to right: song number in the charts, *D*_*N*_, depends only on the rate of turnover of unique entities (songs), and takes no account of their phenotypic similarity. Class diversity, *D*_S_, is the effective number of styles and captures functional diversity. Topic diversity, *D*_T_, is the effective number of musical topics used each year, averaged across the harmonic and timbral topics. Disparity, *D*_Y_, or phenotypic range is estimated as the total standard deviation within a year. Note that although in ecology *D*_S_ and *D*_Y_ are often applied to sets of distinct species or lineages they need not be; our use of them implies nothing about the ontological status of our styles and topics. For full definitions of the diversity measures, see electronic supplementary material, M11. Shaded regions define eras separated by musical revolutions (figure 5).

To test these ideas, we estimated four yearly measures of diversity (figure 4). We found that although all four evolve, two—topic diversity and disparity—show the most striking changes, both declining to a minimum around 1986, but then rebounding and increasing to a maximum in the early 2000s. Because neither of these measures track song number, their dynamics cannot be due to varying numbers of songs in the Hot 100; nor, because our sampling over 50 years is nearly complete, can they be due to the over-representation of recent songs—the so-called pull of the recent [34]. Instead, their dynamics are due to changes in the frequencies of musical styles.

The decline in topic diversity and disparity in the early 1980s is due to a decline of timbral rather than harmonic diversity (electronic supplementary material, figure S1). This can be seen in the evolution of particular topics (figure 2). In the early 1980s timbral topics T1 (drums, aggressive, percussive) and T5 (guitar, loud, energetic) become increasingly dominant; the subsequent recovery of diversity is due to the relative decrease in frequency of the these topics as T3 (energetic, speech, bright) increases. Put in terms of styles, the decline of diversity is due to the dominance of genres such as new wave, disco, hardrock; its recovery is due to their waning with the rise of rap and related genres (figure 2). Contrary to current theories of musical evolution, then, we find no evidence for the progressive homogenization of music in the charts and little sign of diversity cycles within the 50 year time frame of our study. Instead, the evolution of chart diversity is dominated by historically unique events: the rise and fall of particular ways of making music.

### 3.4 Musical evolution is punctuated by revolutions

The history of popular music is often seen as a succession of distinct eras, e.g. the ‘Rock Era’, separated by revolutions [3,6,14]. Against this, some scholars have argued that musical eras and revolutions are illusory [5]. Even among those who see discontinuities, there is little agreement about when they occurred. The problem, again, is that data have been scarce, and objective criteria for deciding what constitutes a break in a historical sequence scarcer yet.

**Figure 5.**
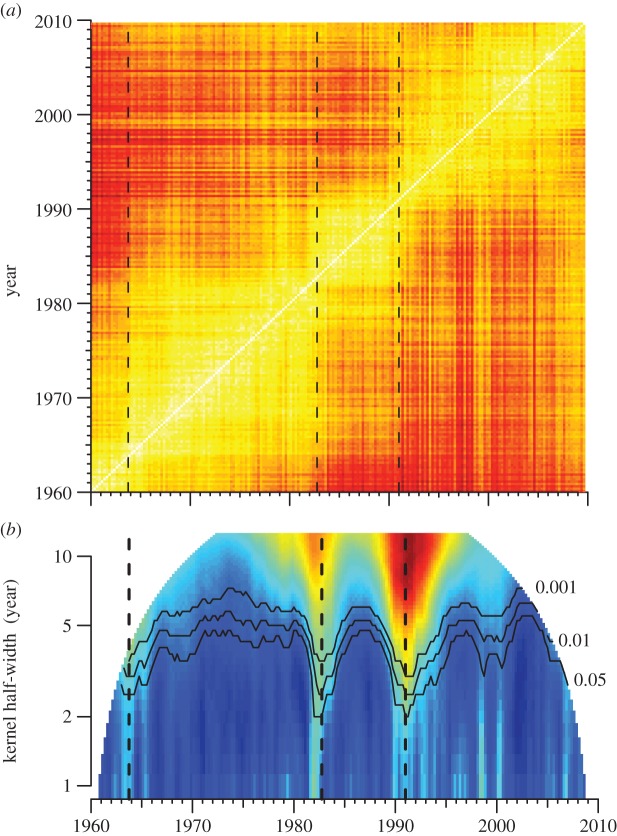
Musical revolutions in the Billboard Hot 100. (*a*) Quarterly pairwise distance matrix of all the songs in the Hot 100. (*b*) Rate of stylistic change based on Foote Novelty over successive quarters for all windows 1–10 years, inclusive. The rate of musical change—slow-to-fast—is represented by the colour gradient blue, green, yellow, red, brown: 1964, 1983 and 1991 are periods of particularly rapid musical change. Using a Foote Novelty kernel with a half-width of 3 years results in significant change in these periods, with Novelty peaks in 1963–Q4 (*p*<0.01), 1982–Q4 (*p*<0.01) and 1991–Q1 (*p*<0.001) marked by dashed lines. Significance cut-offs for all windows were empirically determined by random permutation of the distance matrix. Significance contour lines with *p*-values are shown in black.

To determine directly whether rate discontinuities exist we divided the period 1960–2010 into 200 quarters and used the principal components of the topic frequencies to estimate a pairwise distance matrix between them (figure 5*a*). This matrix suggested that, while musical evolution was ceaseless, there were periods of relative stasis punctuated by periods of rapid change. To test this impression, we applied a method from Music Information Retrieval, Foote Novelty, which estimates the magnitude of change in a distance matrix over a given temporal window [35]. The method relies on a kernel matrix with a checkerboard pattern. Because a distance matrix exposes just such a checkerboard pattern at change points [35], convolving it with the checkerboard kernel along its diagonal directly yields the novelty function (electronic supplementary material, M11). We calculated Foote Novelty for all windows between 1 and 10 years and, for each window, determined empirical significance cut-offs based on random permutation of the distance matrix. We identified three revolutions: a major one around 1991 and two smaller ones around 1964 and 1983 (figure 5*b*). From peak to succeeding trough, the rate of musical change during these revolutions varied four- to six-fold.

This temporal analysis, when combined with our style clusters (figure 3), shows how musical revolutions are associated with the expansion and contraction of particular musical styles. Using quadratic regression models, we identified the styles that showed significant (*p*<0.01) change in frequency against time in the 6 years surrounding each revolution (electronic supplementary material, table S2). We also carried out a style-enrichment analysis for the same periods (electronic supplementary material, table S2). Of the three revolutions, 1964 was the most complex, involving the expansion of several styles—1, 5, 8, 12 and 13—enriched at the time for soul- and rock-related tags. These gains were bought at the expense of styles 3 and 6, both enriched for doowop among other tags. The 1983 revolution is associated with an expansion of three styles—8, 11 and 13—here enriched for new wave-, disco- and hard rock-related tags and the contraction of three styles—3, 7 and 12—here enriched for soft rock-, country-related or soul + r`n'b-related tags. The largest revolution of the three, 1991, is associated with the expansion of style 2, enriched for rap-related tags, at the expense of styles 5 and 13, here enriched for rock-related tags. The rise of rap and related genres appears, then, to be the single most important event that has shaped the musical structure of the American charts in the period that we studied.

### 3.5 The British did not start the American revolution of 1964

**Figure 6.**
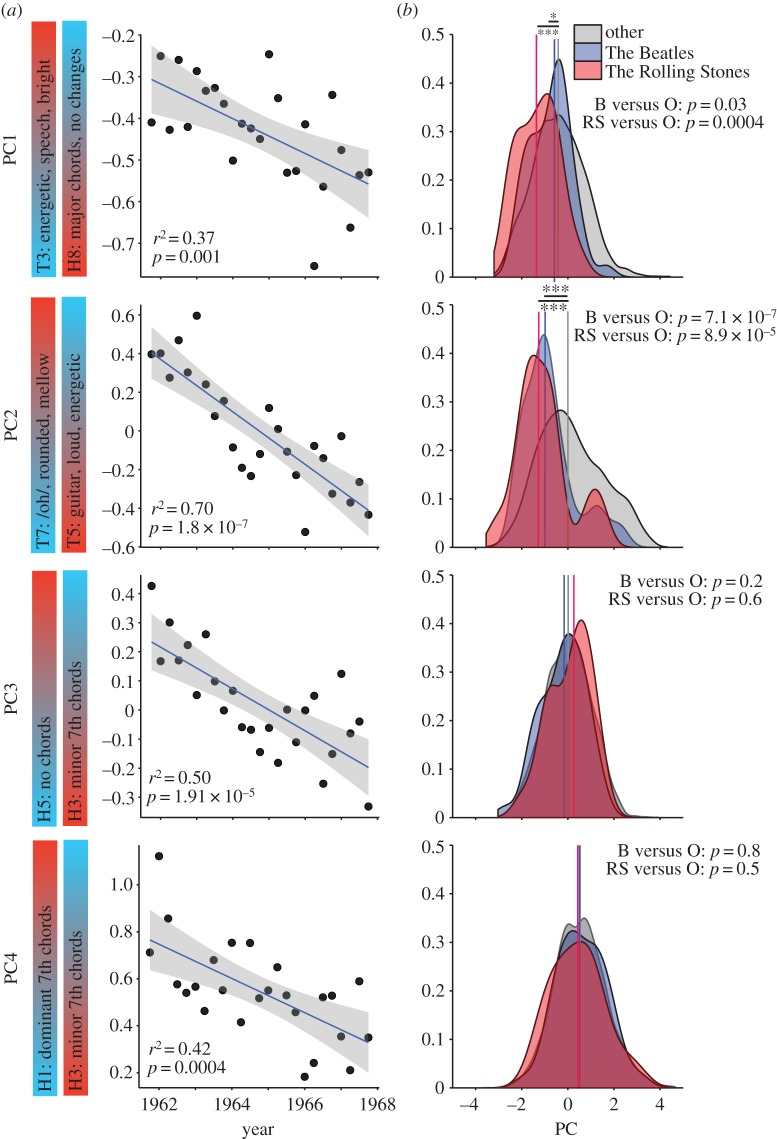
The British Invasion in the American revolution of 1964. Top to bottom: PC1–PC4. (*a*) Linear evolution of quarterly medians of four PCs in the 6 years (24 quarters) flanking 1963–Q4, the peak of the 1964 revolution. The population medians of all four PCs decrease, and these decreases begin well before the start of the British Invasion (BI) in late 1963, implying that BI acts cannot be solely responsible for the changes in musical style evident at the time. For each PC, the two topics that load most strongly are indicated, with sign of correlation—high, red to low, blue—indicated (electronic supplementary material, figure S2). (*b*) Frequency density distributions of four PCs for the Beatles, The Rolling Stones and songs by all other artists around the 1964 revolution. For PC1 and PC2, but not PC3 and PC4, The Beatles and The Rolling Stones have significantly lower median values than the rest of the population, indicated by arrows, implying that these BI artists adopted a musical style that exaggerated existing trends in the Hot 100 towards increased use of major chords and decreased use of ‘bright’ speech (PC1) and increased guitar-driven aggression and decreased use of mellow vocals (PC2). Vertical lines represent medians; *p*-values based on Mann–Whitney–Wilcoxon rank sum test; The Beatles (B): *n*=46; The Rolling Stones (RS): *n*=20; other artists (O): *n*=3114.

Our analysis does not reveal the origins of musical styles; rather, it shows when changes in style frequency affect the musical structure of the charts. Bearing this in mind, we investigated the roles of particular artists in one revolution. On 26 December 1963, The Beatles released *I want to hold your hand* in the USA. They were swiftly followed by dozens of British acts who, over the next few years, flooded the American charts. It is often claimed that this ’British Invasion’ (BI) was responsible for musical changes of the time [36]. Was it? As noted above, around 1964, many styles were changing in frequency; many principal components of the topic frequencies show linear changes in this period too. Inspection of the first four PCs shows that their evolutionary trajectories were all established before 1964, implying that, whereas the British may have contributed to this revolution, they could not have been entirely responsible for it (figure 6*a*). We then compared two of the most successful BI acts, The Beatles and The Rolling Stones, with the rest of the Hot 100 (figure 6*b*). In the case of PC1 and PC2, the songs of both bands have (low) values that anticipate the Hot 100’s trajectory: for these musical attributes, they were literally ahead of the curve. In the case of PC3 and PC4, their songs resemble the rest of the Hot 100: for these musical attributes, they were merely on-trend. Together, these results suggest that, even if the British did not initiate the American revolution of 1964, they did exploit it and, to the degree that they were imitated by other artists, fanned its flames. Indeed, the extraordinary success of these two groups—66 Hot 100 hits between them prior to 1968—may be attributable to their having done so.

## 4. Discussion and Conclusion

Our findings provide a quantitative picture of the evolution of popular music in the USA over the course of 50 years. As such, they form the basis for the scientific study of musical change. Those who wish to make claims about how and when popular music changed can no longer appeal to anecdote, connoisseurship and theory unadorned by data. Similarly, recent work has shown that it is possible to identify discrete stylistic changes in the history of Western classical music by clustering on motifs extracted from a corpus of written scores [29]. Insofar that our approach is based on audio, it can also be applied to music for which no scores exist, including that from pre-Modern cultures [19,37,38]. We have already applied a similar approach to the classification of art music (‘classical music’) into historical periods [39]. More generally, music is a natural starting point for the study of stylistic evolution because it is not only a universal human cultural trait [40] but also measurable, largely determined by form, and available in a relatively standardized format (digital recordings).

Our study is limited in several ways. First, it is limited by the features studied. Our measures must capture only a fraction of the phenotypic complexity of even the simplest song; other measures may give different results. However, the finding that our classifications are supported by listener genre-tags gives us some confidence that we have captured an important part of the perceptible variance of our sample. Second, in confining our study to the Hot 100, 1960–2010, we have only sampled a small fraction of the new singles released in the USA; a complete picture would require compiling a database of several million songs, which in itself is a challenge [41]. Given that the Hot 100 is certainly a biased subset of these songs, our conclusions cannot be extended to the population of all releases. Finally, we are interested in extending the temporal range of our sample to at least the 1940s—if only to see whether 1955 was, as many have claimed, the birth date of Rock’n’Roll [42].

We have not addressed the causes of the dynamics that we detect. Like any cultural artefact—and any living organism—music is the result of a variational-selection process [16–19]. In evolutionary biology, causal explanations of organismal diversity appeal to intrinsic constraints (developmental or genetic), ecological factors (competition among individuals or lineages) and stochastic events (e.g. rocks from space) [43–45]. By analogy, a causal account of the evolution of music must ultimately contain an account of how musicians imitate, and modify, existing music when creating new songs, that is, an account of the mode of inheritance, the production of musical novelty and its constraints. The first of these—inheritance and its constraints—is obscure [46,47]; the second—selection—less so. The selective forces acting upon new songs are at least partly captured by their rise and fall through the ranks of the charts. Many anecdotal histories of music attempt to explain these dynamics. For example, the rise of rap in the charts has been credited to the television show *Yo, MTV Raps!* first broadcast in 1988 [48]. A general, multilevel, selection theory, not restricted to Mendelian inheritance, should provide a means for such hypotheses to be tested [49–51].

Finally, we note that the statistical tools used in this study are quite general. LDA can be used to study the evolving structure of many kinds of assemblages; Foote Novelty can be used to detect rate discontinuities in temporal sequences of distances based on many kinds of phenotypes. Such tools, and the existence of large digital corpora of cultural artefacts—texts, music, images, computer-aided design (CAD) files—now permits the evolutionary analysis of many dimensions of modern culture. We anticipate that the study of cultural trends based upon such datasets will soon constrain and inspire theories about the evolution of culture just as the fossil record has for the evolution of life [52,53].

## Supplementary Material

One file: Supporting Information. Contains detailed methods and supplementary figures

## Data accessibility

All methods and supplementary figures and tables are available in the electronic supplementary material. Extensive data, including song titles, artists, topic frequencies and tags are available from the Figshare repository: main data frame 10.6084/m9.figshare.1309953; secondary data frame 10.6084/m9.figshare.1309950.

## References

[1] Adorno TW 1941 On popular music. Stud. Philos. Soc. Sci. 9, 17–48.

[2] Adorno TW 1975 Culture industry reconsidered. New German Critique 6, 12–19. (doi:10.2307/487650)

[3] Frith S 1988 Music for pleasure. Cambridge, UK: Polity Press.

[4] Mauch M 2011 The anatomy of the UK charts: a light-hearted investigation of 50 years of UK charts using audio data; collection of 5 blog posts. See http://schall-und-mauch.de/anatomy-of-the-charts/.

[5] Negus K 1996 Popular music theory. Cambridge, UK: Polity.

[6] Stanley B 2013 Yeah yeah yeah: the story of modern pop. London: Faber & Faber.

[7] Morrissey SP 2013 Autobiography. London, UK: Penguin.

[8] Peterson RA, Berger DG 1975 Cycles in symbol production. Am. Soc. Rev. 40, 158–173. (doi:10.2307/2094343)

[9] Lopes PD 1992 Innovation and diversity in the popular music industry, 1969–1990. Am. Sociol. Rev. 57, 56–71. (doi:10.2307/2096144)

[10] Christianen M 1995 Cycles in symbol production? A new model to explain concentration, diversity, and innovation in the music Industry. Popular Music 14, 55–93. (doi:10.1017/S0261143000007637)

[11] Alexander PJ 1996 Entropy and popular culture: product diversity in the popular music recording industry. Am. Sociol. Rev. 61, 171–174. (doi:10.2307/2096412)

[12] Peterson RA, Berger DG 1996 Measuring industry concentration, diversity, and innovation in popular music. Am. Sociol. Rev. 61, 175–178. (doi:10.2307/2096413)

[13] Crain WM, Tollinson RD 1997 Economics and the architecture of popular music. J. Econ. Behav. Organ. 901, 185–205. (doi:10.1016/S0167-2681(96)00901-8)

[14] Tschmuck P 2006 Creativity and innovation in the music industry. Dordrecht, The Netherlands: Springer.

[15] Klein CC, Slonaker SW 2010 Chart turnover and sales in the recorded music industry: 1990–2005. Rev. Ind. Organ. 36, 351–372. (doi:10.1007/s11151-010-9250-z)

[16] Leroi AM, Swire J 2006 The recovery of the past. World of Music 48, 43–54.

[17] Jan S 2007 The memetics of music: a Neo-Darwinian view of musical structure and culture. Farnham, UK: Ashgate.

[18] MacCallum RM, Mauch M, Burt A, Leroi AM 2012 Evolution of music by public choice. Proc. Natl Acad. Sci. USA 109, 12 081–12 086. (doi:10.1073/pnas.1203182109) 10.1073/pnas.1203182109 PMC3409751 22711832

[19] Savage PE, Brown S 2013 Toward a new comparative musicology. Anal. Approach. World Music 2, 148–197.

[20] Cavalli-Sforza LL 1981 Cultural transmission and evolution: a quantitative approach. Princeton, NJ: Princeton University Press. 7300842

[21] Boyd R, Richerson PJ 1985 Culture and the evolutionary process. Chicago, IL: University of Chicago Press.

[22] Shennan S 2009 Pattern and process in cultural evolution. Berkley, CA: University of California Press.

[23] Steele J, Jordan P, Cochrane E 2010 Evolutionary approaches to cultural and linguistic diversity. Phil. Trans. R. Soc. B 365, 3781–3785. (doi:10.1098/rstb.2010.0202) 21041203 10.1098/rstb.2010.0202 PMC2981919

[24] Mesoudi A 2011 Cultural evolution: how Darwinian theory can explain human culture and synthesize the social sciences. Chicago, IL: University of Chicago Press.

[25] Whiten A, Hinde RA, Stringer C, Laland KN 2012 Culture evolves. Oxford, UK: Oxford University Press.

[26] Dodds PS, Danforth CM 2010 Measuring the happiness of large-scale written expression: songs, blogs, and presidents. J. Happ. Stud. 11, 441–456. (doi:10.1007/s10902-009-9150-9)

[27] Shanahan D, Broze Y 2012 A diachronic analysis of harmonic schemata in jazz. In *Proc. 12th Int. Conf. on Music Perception and Cognition and the 8th Triennial Conf. of the European Society for the Cognitive Sciences of Music, Thessaloniki, Greece, 23–28 July 2012*, pp. 909–917.

[28] Serrà J, Corral A, Boguñá M, Haro M, Arcos JLI 2012 Measuring the evolution of contemporary Western popular music. Sci. Rep. 2, 521 . (doi:10.1038/srep00521) 22837813 10.1038/srep00521 PMC3405292

[29] Rodriguez Zivic PH, Shifres F, Cecchi GA 2013 Perceptual basis of evolving Western musical styles. Proc. Natl Acad. Sci. USA 110, 10 034–10 038. (doi:10.1073/pnas.1222336110) 10.1073/pnas.1222336110 PMC3683751 23716669

[30] Foster P, Mauch M, Dixon S 2014 Sequential complexity as a descriptor for musical similarity. IEEE Trans. Audio, Speech, Lang. Process. 22, 1965–1977. (doi:10.1109/TASLP.2014.2357676)

[31] Castillo-Davis CI, Hartl DL 2003 GeneMerge – post genomic analysis, data mining, and hypothesis testing. Bioinformatics 19, 891–892. (doi:10.1093/bioinformatics/btg114) 12724301 10.1093/bioinformatics/btg114

[32] Zollo P 2003 Songwriters on songwriting. Jackson, TN: Da Capo Press.

[33] Simpson GG 1944 Tempo and mode in evolution. New York, NY: Columbia University Press.

[34] Jablonski D, Roy K, Valentine JW, Price RM, Anderson PS 2003 The impact of the pull of the recent on the history of bivalve diversity. Science 300, 1133–1135. (doi:10.1126/science.1083246) 12750517 10.1126/science.1083246

[35] Foote J 2000 Automatic audio segmentation using a measure of audio novelty. In *IEEE Int. Conf. on Multimedia and Expo., New York, NY, 30 July–2 August 2000,* vol. 1, pp. 452–455.

[36] Fitzgerald J 1995 When the Brill building met Lennon–McCartney: continuity and change in the early evolution of the mainstream pop song. Popular Music Soc. 19, 59–77. (doi:10.1080/03007769508591581)

[37] Lomax A 1968 Folk song style and culture. Washington, DC: American Association for the Advancement of Science.

[38] Lomax A, Berkowitz N 1972 The evolutionary taxonomy of culture. Science 177, 228–239. (doi:10.1126/science.177.4045.228) 17815614 10.1126/science.177.4045.228

[39] Weiß C, Mauch M, Dixon S 2014 Timbre-invariant audio features for style analysis of classical music. In *Proc. 11th Music Computing Conf. (SMC 2014), Athens, Greece, 14–20 September 2014*, pp. 1461–1468.

[40] Brown DE 1991 Human universals. Philadelphia, PA: Temple University Press.

[41] Bertin-Mahieux T, Ellis DPW, Whitman B, Lamere P 2011 The million song dataset. In *Proc. 12th Int. Soc. Music Information Retrieval Conf. (ISMIR 2011), Miami, FL, 24–28 October 2011*, pp. 591–596.

[42] Peterson RA 1990 Why 1955? Explaining the advent of rock music. Popular Music 9, 97–116. (doi:10.1017/S0261143000003767)

[43] Erwin DH 2007 Disparity: morphological pattern and developmental context. Palaeontology 50, 57–73. (doi:10.1111/j.1475-4983.2006.00614.x)

[44] Gould SJ 2002 The structure of evolutionary theory. Cambridge, MA: Harvard University Press.

[45] Jablonski D 2008 Species selection: theory and data. Annu. Rev. Ecol. Syst. 39, 501–524. (doi:10.1146/annurev.ecolsys.39.110707.173510)

[46] Pachet F 2006 Creativity studies and musical interaction. In *Musical creativity: multidisciplinary research in theory and practice* (eds I Deliège, GA Wiggins), pp. 347–358. Hove, UK: Psychology Press.

[47] McIntyre P 2008 Creativity and cultural production: a study of contemporary Western popular music songwriting. Creat. Res. J. 20, 40–52. (doi:10.1080/10400410701841898)

[48] George N 2005 Hip hop America. London, UK: Penguin.

[49] Price GR 1970 Selection and covariance. Nature 227, 520–521. (doi:10.1038/227520a0) 5428476 10.1038/227520a0

[50] Price GR 1972 Extension of covariance selection mathematics. Ann. Hum. Genet. 35, 485–490. (doi:10.1111/j.1469-1809.1957.tb01874.x) 5073694 10.1111/j.1469-1809.1957.tb01874.x

[51] Frank SA 1995 George Price’s contributions to evolutionary genetics. J. Theor. Biol. 175, 373–388. (doi:10.1006/jtbi.1995.0148) 7475081 10.1006/jtbi.1995.0148

[52] Michel J 2011 Quantitative analysis of culture using millions of digitized books. Science 331, 176–182. (doi:10.1126/science.1199644) 21163965 10.1126/science.1199644 PMC3279742

[53] Perc M 2012 Evolution of the most common English words and phrases over the centuries. J. R. Soc. Interface 9, 3323–3328. (doi:10.1098/rsif.2012.0491) 22832364 10.1098/rsif.2012.0491 PMC3481586

