# Supplementary material for: The evolution of popular music: USA 1960–2010
Source: R Soc Open Sci. 2015 May 6;2(5):150081. doi: 10.1098/rsos.150081 (PMC4453253; doi:10.1098/rsos.150081)
Supplement: One file: Supporting Information. Contains detailed methods and supplementary figures [file rsos150081supp1.pdf]

The evolution of popular music: USA 1960-2010: Supporting Information

Matthias Mauch, Robert M. MacCallum, Mark Levy, Armand M. Leroi

15 October, 2014

Contents

M Materials and Methods **2**

M.1 The origin of the songs . . . 2
M.2 Measuring Harmony. . . . 2
M.3 Measuring Timbre. . . . 3
M.4 Making musical lexica . . . 3
M.5 Semantic lexicon annotation . . . 4
M.6 Topic extraction . . . 5
M.7 Semantic topic annotations . . . 6
M.8 User-generated tags . . . 7
M.9 Identifying musical Styles clusters: k-means and silhouette scores . . . 7
M.10 Diversity metrics . . . 8
M.11 Identifying musical revolutions . . . 9
M.12 Identifying Styles that change around each revolution . . . 10

S Supplementary Text & Tables **11**

M Materials and Methods

M.1 The origin of the songs

Metadata on the complete Billboard Hot 100 charts were obtained through the (now defunct) Billboard API, consisting of artist name, track name, and chart position in every week of the charts from 1957 to early 2010. We use only songs from 1960 through 2009 since these years have complete coverage. Using a Last.fm's proprietary matching procedure, we associated Last.fm MP3 audio recordings with the chart entries. Each recording is 30 seconds long. We use 17,094 songs, covering 86% of the weekly Billboard charts (84% before 2000, 95% from 2000 onward (figure M1). This amounts to 69% of unique audio recordings. The total duration of the music data is 143 hours.

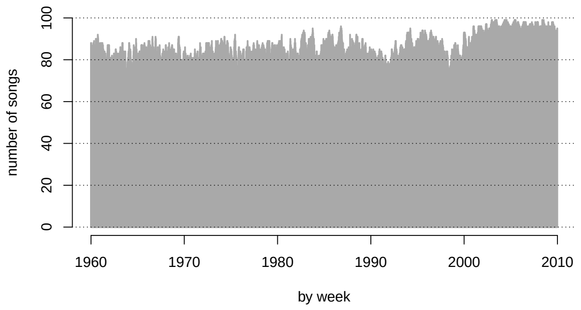

Figure M1: Coverage of the Billboard Hot 100 Charts by week.

To validate our impression that data quality was good, a random sub-sample of 9928 songs was vetted by hundreds of volunteers recruited on the internet. The participants were presented with two recordings, and for each were asked to to answer the question "Does recording [...] have very poor audio quality?". We analysed those 5593 recordings that were judged at least twice. A recording was considered poor quality if it was marked as such by a majority vote. Overall, this was the case in only 3.8% of the recordings, with a bias towards worse quality recordings in the 1960s (9.1%; 1970 and later: 1.8%). To examine the effect of bad songs, we removed them and compared the estimated mean q of each topic (Section M.6) for the total population versus the population of 'good' songs for each year of the 1960s. In no case did we find that they were significantly different. We conclude that recording quality will have a negligible effect on our results.

All songs were decoded to PCM WAV format (44100 Hz, 16 bit). The songs were then band-pass-filtered using the Audio Degradation Toolbox [1] to reduce differences in recording equalisation in the bass and high treble frequencies (stop-band frequencies: 67 Hz, 6000 Hz).

M.2 Measuring Harmony.

The harmony features consist of 12-dimensional chroma features (also: pitch class profiles) [2]. Chroma is widely used in MIR as a robust feature for chord and key detection [3], audio thumbnailing [4], and automatic structural segmentation [5]. In every frame chroma represents the activations (i.e. the strength)

$$\mathbf{c} = (c_1, \ldots, c_{12})$$

corresponding to the 12 pitch classes in the chromatic musical scale (i.e. that of the piano): A, B♭, B C, . . . , G, A♭. We use the NNLS Chroma implementation [6] to extract chroma at the same frame rate as the timbre features (step size: 1024 samples = 23ms, i.e. 43 per second), but with the default frame size

of 16384 samples. The chroma representation (often called chromagram) of the complete 30 s excerpt of "Bohemian Rhapsody" is shown in figure 1 (main text).

M.3 Measuring Timbre.

The timbre features consist of 12 Mel-frequency cepstral coefficients (MFCCs), one delta-MFCC value, and one Zero-crossing Count (ZCC) feature. MFCCs are spectral-domain audio features for the description of timbre and are routinely used in speech recognition [7] and Music Information Retrieval (MIR) tasks [8]. For every frame, they provide a low-dimensional parametrisation of the overall shape of the signal's Mel-spectrum, i.e. a spectral representation that takes into account human near-logarithmic perception of sound in magnitude (log-magnitude) and frequency (Mel scale). We use the first 12 MFCCs (excluding the 0th component) and additionally one delta-MFCC, calculated as the difference between any two consecutive values of the 0th MFCC component. The MFCCs were extracted using a plugin from the Vamp library (link retrieved 27.03.2014) with the default parameters (block size: 2048 samples = 46ms, step size: 1024 samples = 23ms). This amounts to ≈ 43 frames per second. The ZCC (also: zero-crossing rate, ZCR) is a time-domain audio feature which has been used in speech recognition [9] and has been applied successfully to discern drum sounds [10]. It is calculated by simply counting the number of times consecutive samples in a frame are of opposite sign. ZCC is high for noisy signals and transient sounds at the onset of consonants and percussive events. To extract the ZCC we also used a Vamp plugin, extracting features at the same frame rate (43 per second, step size: 1024 sampes = 23ms), but with a block size of 1024 samples. MFCCs and zero crossing counts of "Bohemian Rhapsody" are shown in figure 1 (main text).

M.4 Making musical lexica

Since we aim to apply topic models to our data (see Section M.6), we need to discretise our raw features into musical lexica. We have one timbral lexicon (T-Lexicon) and one harmonic lexicon (H-Lexicon).

Timbre.

In order to define the T-Lexicon we followed an unsupervised feature learning approach by quantising the feature space into 35 discrete classes as follows. First, we randomly selected 20 frames from each of 11350 randomly selected songs (227 from every year), a total of 227,000 frames. The features were then standardised, and de-correlated using principal component analysis (PCA). The PCA components were once more standardised. We then applied model-based clustering (Gaussian mixture models, GMM) to the standardised de-correlated data, using the built-in Matlab function gmdistribution.fit with full covariance matrix [11]. The GMM with 35 mixtures (clusters) was chosen as it minimised the Bayes Information Criterion. We then transformed all songs according to the same PCA, scaling and cluster mapping transformations. In particular, every audio frame was assigned to its most likely cluster according to the GMM. Frames with cluster probabilities of < 0.5 were removed.

Harmony.

Our H-Lexicon consists of all 192 possible changes between the most frequently used chord types in popular music [12]: major (M), minor (m), dominant 7 (7) and minor 7 chords (m7). We use chord changes because they offer a key-independent way of describing the temporal dynamics of harmony. As a chord is defined by its root pitch class (A,Bb,B,C,...,Ab♭) and its type, our system gives rise to $4 \times 12 = 48$ chords. Each of the chords can be represented as a binary chord template with 12 elements corresponding to the twelve pitch classes. For example, the four chords with root A are these.

$$CT_{AM} = (1, 0, 0, 0, 1, 0, 0, 1, 0, 0, 0, 0)$$

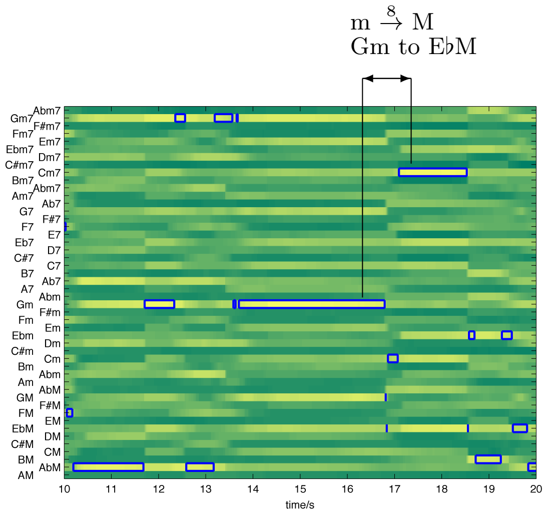

Figure M2: Chord activation, with the most salient chords at any time highlighted in blue. Excerpt of "Bohemian Rhapsody" by Queen.

$$CT_{Am} = (1,0,0,1,0,0,0,1,0,0,0,0)$$
$$CT_{A7} = (1,0,0,0,1,0,0,1,0,0,1,0)$$
$$CT_{Am7} = (1,0,0,1,0,0,0,1,0,0,1,0)$$

At every frame we estimate the locally most likely chord by correlating the chroma vectors (Section M.2) with the binary chord templates (see, e.g. [13]), i.e. a given chroma frame $\mathbf{c} = (c_1, \ldots, c_{12})$ is correlated to a chord template $CT = (CT_1, \ldots, CT_{12})$ by using Pearson's rho,

$$\rho_{CT,\mathbf{c}} = \sum_{i=1}^{12} \frac{(CT_i - \overline{CT})(c_i - \overline{\mathbf{c}})}{\sigma_{CT}\sigma_{\mathbf{c}}},$$

where the bar over variables denotes the sample mean and $\sigma_{\cdot}$ denotes the sample standard deviation of the corresponding vector. To smooth these correlation scores over time, we apply a median filter of length 43 (1 second). An example of the resulting smoothed chord activation matrix is shown in figure M2. We then choose the chord with the highest median-smoothed correlation and combine the two chord labels spaced 1 second apart into one chord change label, retaining only the relative root positions of the chords and both chord types [14], as demonstrated in figure M2. If the chord change was ambiguous (mean correlation of the two chords < 0.4), the chord change label was set to an additional 193rd label *NA*.

In summary, we have obtained two lexica of frame-wise discrete labels, one for timbre (35 classes) and one for harmony (193 chord changes). Each allows us to describe a piece of music as a count vector giving counts of timbre classes and chord changes, respectively.

M.5 Semantic lexicon annotation

Since we can now express our music in terms of lexica of discrete items, we can attach human-readable labels to these items. In the case of the 193 chord changes (H-Lexicon), an intrinsic musical interpretation exists. The most frequent chord changes are given in additional online tables (Section 3), along with some explanations and counts over the whole corpus.

The 35 classes in the T-Lexicon do not have *a priori* interpretations, so we obtained human annotations on a subset of our data. First, we randomly selected 100 songs, two from each year, and concatenated

the audio that belonged to the same of the 35 sound classes from our T-Lexicon using an overlap-add approach. That is, each audio file contained frames from only one of the timbre classes introduced in Section M.4, but from up to 100 songs. The resulting 35 sound class files can be accessed on SoundCloud[1]). We noticed that each of the files does indeed have a timbre characteristic; some captured a particular vowel sound, others noisy hi-hat and crash cymbal sounds, others again very short, percussive sounds. We then asked ten human annotators to individually describe these sounds. Each annotator listened to all 35 files and, for each, subjectively chose 5 terms that described the sound from a controlled vocabulary consisting of the following 34 labels manually compiled from initial free-vocabulary annotations:

mellow, aggressive, dark, bright, calm, energetic, smooth, percussive, quiet, loud, harmonic, noisy, melodic, rounded, harsh, vocal, instrumental, speech, instrument: drums, instrument: guitar, instrument: piano, instrument: orchestra, instrument: male voice, instrument: female voice, instrument: synthesiser, 'ah', 'ay', 'ee', 'er', 'oh', 'ooh', 'sh', 'ss', [random – I find it hard to judge].

On average, the most agreed-upon label per class was chosen by 7.5 (mean) of the 10 annotators, indicating good agreement. Even the second- and third-ranking labels were chosen by more than half of the annotators (means 6.4 and 5.68). Figure M3 shows the agreement of the top labels from rank 1 to 8.

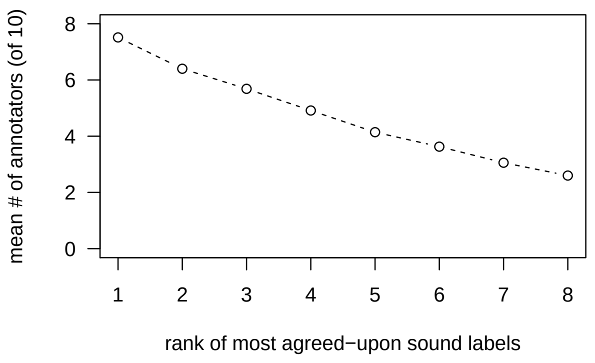

Figure M3: Agreement of the 10 annotators in the semantic sound annotation task.

M.6 Topic extraction

For timbre and harmony separately, a topic model is estimated from the song-wise counts, using the implementation of Latent Dirichlet Allocation (LDA) [15] provided in the `topicmodels` library [16] for R. LDA is a hierarchical generative model of a corpus. The original model was formulated in the context of a text corpus in which

a) every document (here: song) is represented as a discrete distribution over N_T topics

b) every every topic is represented as a discrete distribution over all possible words (here: H-Lexicon or T-Lexicon entries)

Since the T- and H-Lexicon count vectors introduced in Section M.4 are of the same format as word counts, we can apply the same modelling procedure. That is, by means of probabilistic inference on the model, the LDA method estimates the topic distributions of each song (probabilities of a song using a particular topic) and the topics' lexical distribution (probabilities over the H- and T-lexica) from the lexicon count vectors.

We used the LDA function, which implements the variational expectation-maximization (VEM) algorithm to estimate the parameters, setting the number of topics to 8. Hence, we obtained one model with

[1] https://soundcloud.com/descent-of-pop/sets/cluster-sounds

8 T-Topics, and one with 8 H-Topics. Topic models allow us to encode every song as a distribution over T- and H-Topics,

$$\mathbf{q}^T = (q_1^T, q_2^T, \ldots, q_8^T)$$
$$\mathbf{q}^H = (q_1^H, q_2^H, \ldots, q_8^H)$$

The probabilities can be interpreted as the proportion of frames in the song belonging to the topic. When it is clear from the context which T- or H-Topic we are concerned with we denote these by the letter q, and their mean over a group of songs by $\bar{q}$. Mean values by year for all topics are shown in figure 2 in the main text with 95% confidence intervals based on quantile bootstrapping.

In the same manner, we calculate means and bootstrap confidence intervals for all artists with at least 10 chart entries and all Last.fm tags (introduced in Section M.8) with at least 200 occurrences. The artists with the highest and lowest mean q of each topic and the respective listing of tags can be found in additional online tables (Sections 1 and 2).

M.7 Semantic topic annotations

In this section we show how to map the semantic interpretations of our harmony and timbre lexica (see Section M.5) to the 8 T-Topics and 8 H-Topics. This allows us to work with the topics rather than the large number of chord changes and sound classes.

Harmony.

Each H-Topic is defined as a distribution $P(E_i^H)$ over all H-lexicon entries E_i^H, $i = 1, \ldots, 193$ (the 193 different chord changes). The most most probable chord changes for each topic can be found in our additional online tables (Section 3). For example, the most likely chord change in H-Topic 4 is a Major chord followed by another Major chord 7 semitones higher, e.g. C to G. The interpretation of a topic, then, is the coincidence of such chord changes in a piece of music. Interpretations of the 8 H-Topics can be found in Table 1.

Timbre.

In order to obtain interpretations for the T-Topics we map the semantic annotations of the T-lexicon (Section M.5) to the topics. The semantic annotations of the T-lexicon come as a matrix of counts $W^* = (w_{ij}^*)$ of annotation labels $j = 1, \ldots, 34$ for each of the sound classes $i = 1, \ldots, 35$. We first normalise the columns $w_{\cdot,j}^*$ by root-mean-square normalisation to obtain a scaled matrix W_{ij} with the elements

$$w_{ij} = \frac{w_{ij}^*}{\sqrt{(1/34)\sum_i (w_{ij}^*)^2}}. \tag{1}$$

The matrix $W = (w_{ij})$ expresses the relevance of the jth label for the ith sound class. Since T-Topics are compositions of sound classes, we can now simply map these relevance values to the topics by multiplication. The weight L_j of the jth label for a T-Topic in which sound class E_i^T appears with probability $P(E_i^T)$ is

$$L_j = \sum_{i=1}^{35} w_{ij} P(E_i^T). \tag{2}$$

The top 3 labels for each T-Topic can be found in Table 1.

Table 1: Topic interpretations.

harmonic topics	
H1	changes involving dominant 7th chords
H2	natural minor key changes
H3	changes involving minor 7th chords
H4	simple diatonic changes used in major keys
H5	unrecognised changes or no chordal content
H6	stepwise changes indicating modal harmony
H7	ambiguous major/minor attribution
H8	sustained major chords

timbral topics	
T1	drums, aggressive, percussive
T2	calm, quiet, mellow
T3	energetic, speech, bright
T4	piano, orchestra, harmonic
T5	guitar, loud, energetic
T6	ay, male voice, vocal
T7	oh, rounded, mellow
T8	female voice, melodic, vocal

M.8 User-generated tags

The Last.fm recordings are also associated with tags, generated by Last.fm users, which we obtained via a proprietary process. The tags are usually genre-related (POP, SOUL), but a few also contain information about the instrumentation (e.g. PIANO), feel (e.g. SUMMER), references to particular artists and others. We removed references to particular artists and joined some tags that were semantically identical. After the procedure we had tags for 16085 (94%) of the songs, with a mean tag count of 2.7 per song (median: 3, mode: 4).

M.9 Identifying musical Styles clusters: k-means and silhouette scores

In order to identify musical styles from our data measurements, we first used the 17094×16 (i.e. songs $\times$ topics) data matrix of all topic probabilities $\mathbf{q}^T$ and $\mathbf{q}^H$, and de-correlated the data using PCA (see also figure S2). The resulting data matrix has 14 non-degenerate principal components, which we used to cluster the data using k-means clustering. We chose a cluster number of 13 based on analysing of the mean silhouette width [17] over a range of $k = 2, \ldots, 25$ clusters, each started with 50 random initialisations. The result of the best clustering at $k = 13$ is chosen, and each song is thus classified to a style $s \in \{1, \ldots, 13\}$ (figure M4).

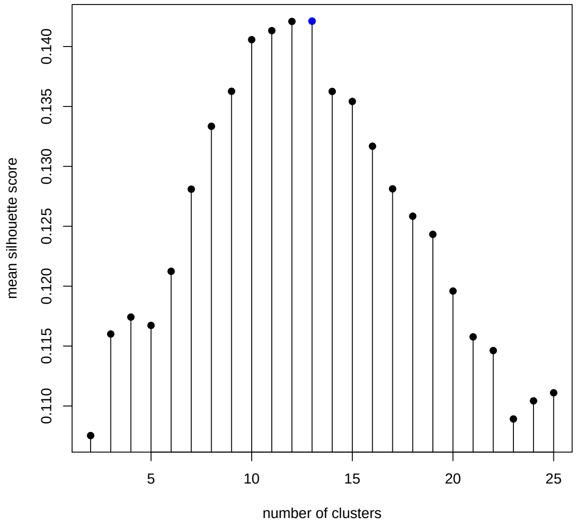

Figure M4: Mean silhouette scores. The optimal number of clusters, $k = 13$ is highlighted in blue.

M.10 Diversity metrics

In order assess the diversity of a set of songs (usually the songs having entered the charts in a certain year) we calculate four different metrics: number of songs (D_N), effective number of styles (style diversity, D_C), effective number of topics (topic diversity, D_T) and disparity (total standard deviation, D_Y). The following paragraphs explain these metrics.

Number of songs.

The simplest measure of complexity is the number of songs D_N. We use it to show that other diversity metrics are not intrinsically linked to this measure.

Effective number of Styles.

In the ecology literature, diversity refers to the effective number of species in an ecosystem. Maximum diversity is achieved when the species' frequencies are all equal, i.e. when they are uniformly distributed. Likewise, minimum diversity is assumed when all organisms belong to the same species. According to [18], diversity for a population of N_s species can formally be defined as

$$D_S = \exp\left(-\sum_{i=1}^{N_s} s_i \ln s_i\right) \tag{3}$$

where s_i, $i = 1, \ldots, N_s$ represents the relative frequency distribution over the N_s species such that $\sum_i s_i = 1$. In particular, the maximum value assumed when all species' relative frequencies are equal is $D = N_s$. If, on the other hand, only one species remains, and all others have frequencies of zero, then $D = 1$, the minimum value.

We use this exact definition to describe the year-wise diversity of acoustical Style clusters in our data (recall that each song has only one Style, but a mixture of Topics). For every year we calculate

the proportion songs s_i, $i = 1, \ldots, 13$ belonging to each of the 13 Styles, and hence we use $N_s = 13$ to calculate $D_S \in [1, 13]$.

Effective number of Topics.

The probability q of a certain topic in a song (see Section M.6) provides an estimate of the proportion of frames in a song that belong to that topic. By averaging over the year, we can get an estimate of the proportion $\bar{q}$ of frames in the whole year, i.e. for all T- and H-Topics we obtain the yearly measurements

$$\bar{\mathbf{q}}^T = (\bar{q}_1^T, \bar{q}_2^T, \ldots, \bar{q}_8^T)$$
$$\bar{\mathbf{q}}^H = (\bar{q}_1^H, \bar{q}_2^H, \ldots, \bar{q}_8^H).$$

Figuratively, we throw all audio frames of all songs into one big bucket pertaining to a year, and estimate the proportion of each topic in the bucket. From these yearly estimates of topic frequencies we can now calculate the effective number of T- and H-Topics in the same way we calculated the effective number of Styles (figure S1).

$$D_T^T = \exp\left(-\sum_{i=1}^{8} \bar{q}_i^T \ln \bar{q}_i^T\right) \tag{4}$$

$$D_T^H = \exp\left(-\sum_{i=1}^{8} \bar{q}_i^H \ln \bar{q}_i^H\right) \tag{5}$$

$$D_T := \frac{D_T^T + D_T^H}{2}, \tag{6}$$

where we define D_T as the overall measure of topic diversity. D_T is shown in the main manuscript (figure 4). The individual H- and T-Topic diversities D_T^T and D_T^H are provided in figure S1. It is evident that the significant diversity decline in the 1980s is mainly due to a decline in timbral topic diversity, while harmonic diversity shows no sign of sustained decline.

Disparity.

In contrast to diversity, disparity corresponds to morphological variety, variety of measurements. Two ecosystems of equal diversity can have different disparity, depending on the extent to which the phenotypes of species differ. A variety of measures, such as average pairwise character dissimilarity and the total variance (sum of univariate variance) [19, 20] have been used to measure disparity. We adopt the square root of total variance, a metric called *total standard deviation* [21, p. 37] as our measure of disparity, i.e. given a set of N observations on T traits as a matrix $X = (x_{n,m})$, we define it as

$$D_Y = \sqrt{\sum_{t=1}^{T} \mathrm{Var}(x_{\cdot,m})}. \tag{7}$$

We apply our disparity measure D_Y to the 14-dimensional matrix of principal components (derived from the topics, as described in Section M.9).

M.11 Identifying musical revolutions

In order to identify points at which the composition of the charts significantly changes, we employ Foote novelty detection [22], a technique often used in MIR [23]. First we pool the 14-dimensional principal component data (see Section M.9) into quarters by their first entry into the charts (January-March, April-June, July-September, October-December) using the quarterly mean of each principal component. We

then construct a matrix (see figure 5 in main text) of pairwise distances between each quarter. Foote's method consists of convolving such a distance matrix with a so-called checkerboard kernel along the main diagonal of the matrix. Checkerboard kernels represent the stylised case of homogeneity within regions (low values in the upper right and bottom-left quadrants) and dissimilarity between regions (high values in the other two quadrants). In such a situation, i.e. when one homogenous era transitions to another, the convolution results in high values.

A kernel with a half-width of 12 quarters (3 years) compares the 3 years prior to the current quarter to those following the current quarter (figure M5). We follow Foote [22] in using checkerboard kernels with Gaussian taper (standard deviation: 0.4 times the half-width). The kernel matrix entries corresponding to the central, "current" quarter are set to zero.

Many different kernel widths are possible. Figure 5B in the main text shows the novelty score for kernels with half-widths between 4 quarters (1 year) and 50 quarters (12.5 years). We can clearly make out three major 'revolutions' (early 1960s, early 1980s, early 1990s) that result in high novelty scores for a wide range of kernel sizes.

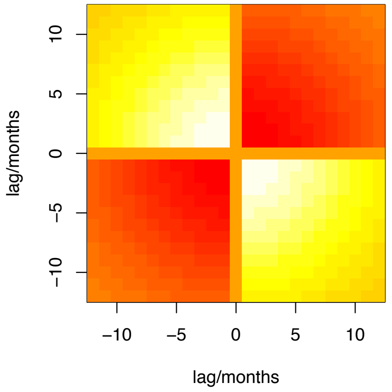

Figure M5: Foote checkerboard kernel for novelty detection.

In order to be able to assess the significance of these regions we compared their novelty scores against novelty values obtained from randomly permuted distance matrices. We first repeated the process 1000 times on distance matrices with randomly permuted quarters. For every kernel size we then calculated the quantiles at confidence levels $\alpha = 0.95$, 0.99 and 0.999. The results are shown as contour lines in figure 5B in the main text.

For further analysis we choose the time scale depicted with a half-width lag of 12 quarters (3 years). This results in three change regions at confidence $p < 0.01$. The 'revolution' points are the points of maximum Foote novelty within the three regions of significant change.

M.12 Identifying Styles that change around each revolution

To identify the styles (clusters) that change around each revolution, we obtained the frequencies of each style for the 24 quarters flanking the peak of a revolution, and estimated the rate of change per annum by a quadratic model. We then used a tag-enrichment analysis to identify those tags associated with each style just around each revolution, see Table S2.

S Supplementary Text & Tables

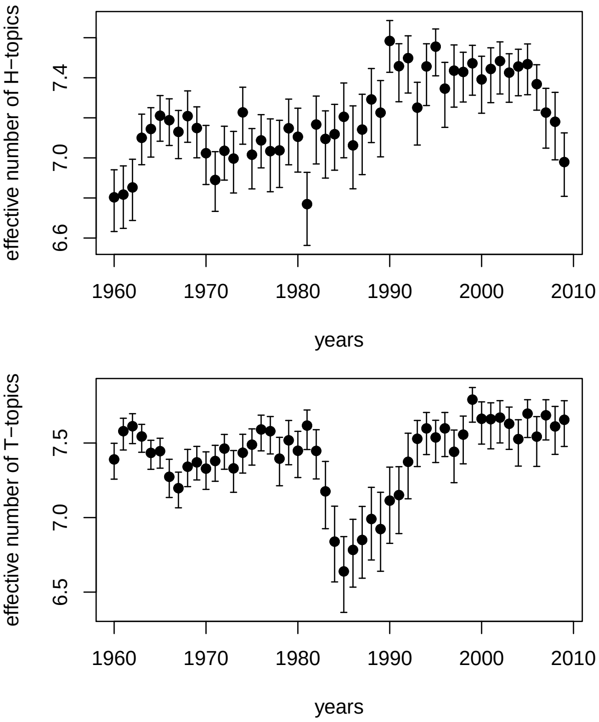

Figure S1: Evolution of Topic diversity. Year-wise topic diversity measures: A D_T^T; B D_T^H.

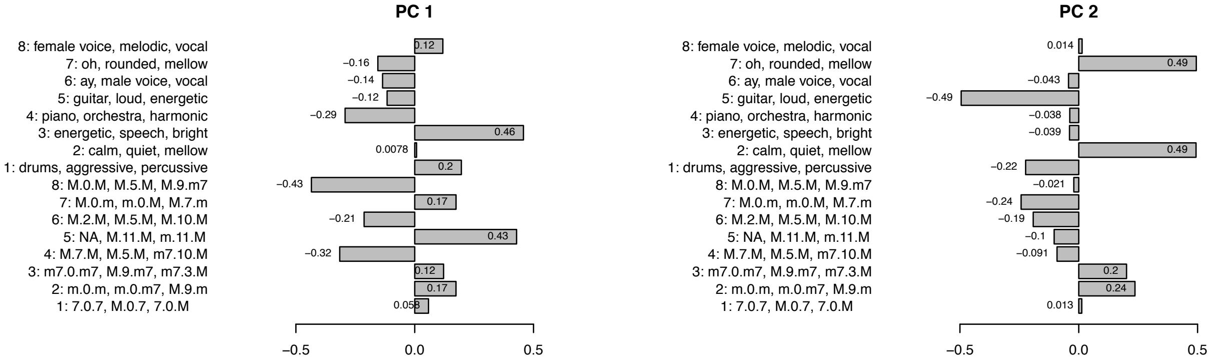

Figure S2: Loadings of the first 2 principal components extracted from the Topic probabilities (see Section M.9).

Table S1: Enrichment analysis: Last.fm user tag over-representation for all Styles over the complete data set (only those with $P < 0.05$)

Style 1	Style 2	Style 3	Style 4	Style 5	Style 6	Style 7
northernsoul	hiphop	easylistening	funk	rock	femalevocal	country
soul	rap	country	blues	classicrock	pop	classiccountry
hiphop	gangstarap	lovesong	jazz	pop	rnb comb	folk
dance	oldschool	piano	soul	newwave	motown	rockabilly
rap	dirtysouth	ballad	instrumental	garage	soul	southernrock
vocaltrance	dance	classiccountry	rocknroll comb	hardrock	glee	
house	dancehall	jazz	northernsoul	garagerock	soundtrack	
	westcoast	doowop	rockabilly	british	musical	
	party	swing				
	comedy	lounge				
	reggae	malevocal				
	newjackswing	50s				
	classic	singersongwriter				
	sexy	romantic				
	urban	softrock				
		christmas				
		acoustic				

Style 8	Style 9	Style 10	Style 11	Style 12	Style 13
dance	classicrock	lovesong	funk	soul	rock
newwave	country	slowjams	blues	rnb comb	hardrock
pop	rock	soul	dance	funk	alternative
electronic	singersongwriter	folk	bluesrock	disco	classicrock
synthpop	folkrock	rnb comb	newwave	slowjams	alt indi rock comb
freestyle	folk	neosoul	electronic	dance	hairmetal
rock	pop	femalevocal	synthpop	neosoul	poppunk
eurodance	softrock	singersongwriter	hiphop	newjackswing	punkrock
newjackswing		acoustic	hardrock	smooth	punk
triphop		romantic		oldschoolsoul	poprock
funk		mellow			metal
		easylistening			powerpop
		jazz			emo
		beautiful			numetal
		smooth			heavymetal
		softrock			glamrock
		ballad			grunge
					british

Table S2: Identifying Styles that change around each revolution and the associated over-represented genre tags in the 24 quarters flanking the revolutions.

revo-lution	style cluster	estim. (linear)	p	estim. (quad.)	p	tags
1964	1	0.044	0.019	0.050	0.009	northernsoul, motown, soul, easylistening, jazz
	2	-0.014	0.291	0.008	0.528	comedy, funny, jazz, easylistening
	3	-0.177	0.000	0.014	0.631	easylistening, jazz, swing, lounge, doowop
	4	-0.060	0.118	-0.009	0.806	jazz, northernsoul, soul, instrumental, blues
	5	0.060	0.014	-0.041	0.081	garagerock, garage, british, psychedelic
	6	-0.171	0.000	-0.045	0.282	femalevocal, motown, northernsoul, soul, doowop
	7	-0.018	0.495	-0.003	0.908	british, folk, surf, malevocal, rocknroll comb
	8	0.062	0.000	0.010	0.441	garagerock, instrumental, northernsoul, surf, soul
	9	0.083	0.014	0.006	0.861	rocknroll comb, northernsoul, motown, soul, garage
	10	-0.020	0.349	0.034	0.109	folk, easylistening, jazz, swing
	11	0.037	0.131	-0.022	0.349	garagerock, blues, soul, northernsoul, instrumental
	12	0.043	0.001	0.000	0.996	northernsoul, soul, motown
	13	0.121	0.000	-0.005	0.821	psychedelic, garagerock, psychedelicrock, motown, british
1983	1	0.031	0.350	-0.050	0.136	newwave, synthpop, disco
	2	0.024	0.189	0.010	0.569	oldschool, funk, comedy
	3	-0.112	0.000	0.021	0.426	lovesong, softrock, easylistening, romantic
	4	-0.022	0.448	-0.005	0.846	newwave, disco, progressiverock
	5	0.020	0.614	-0.019	0.617	newwave, rock, classicrock, progressiverock, pop, synthpop
	6	-0.013	0.598	-0.005	0.828	femalevocal, disco, pop, reggae, musical, soundtrack
	7	-0.098	0.003	0.015	0.598	newwave, classiccountry, softrock, newromantic, rock, southernrock
	8	0.173	0.000	-0.029	0.190	newwave, pop, rock, disco, synthpop
	9	-0.065	0.057	0.065	0.055	classicrock, rock, softrock, progressiverock, newwave, pop
	10	-0.047	0.110	-0.004	0.882	lovesong, softrock
	11	0.048	0.089	-0.058	0.043	newwave, synthpop, rock, classicrock, hardrock
	12	-0.095	0.001	0.011	0.664	funk, disco, soul, smoothjazz, dance
	13	0.150	0.000	0.034	0.236	rock, classicrock, hardrock, newwave, progressiverock, southernrock, powerpop, heavymetal, hairmetal
1991	1	0.034	0.161	-0.018	0.457	house, freestyle, synthpop, newwave, electronic, dance
	2	0.325	0.000	0.043	0.187	hiphop, rap, oldschool, newjackswing, gangstarap, eurodance, westcoast, dance
	3	0.056	0.011	0.011	0.596	ballad, lovesong
	4	0.005	0.822	-0.034	0.153	dance, hairmetal, hardrock, metal, newjackswing
	5	-0.085	0.003	0.003	0.901	rock, hardrock, hairmetal, pop, freestyle
	6	0.000	0.999	0.015	0.344	femalevocal, pop, slowjams, dance, rnb comb, ballad
	7	0.023	0.307	0.007	0.742	rock, hardrock, hairmetal
	8	-0.023	0.584	-0.023	0.576	dance, newjackswing, freestyle, pop, electronic, synthpop, newwave, australian
	9	-0.015	0.690	0.046	0.224	rock, pop, softrock, ballad, hardrock
	10	0.042	0.235	0.008	0.813	slowjams, lovesong, rnb comb
	11	-0.065	0.016	0.030	0.248	newwave, dance, synthpop, hairmetal, rock, freestyle
	12	-0.035	0.447	-0.017	0.712	newjackswing, rnb comb, dance, slowjams, house, pop, soul
	13	-0.207	0.000	-0.112	0.023	hardrock, hairmetal, rock, classicrock, metal, heavymetal, thrashmetal, madchester

References

[1] Mauch M, Ewert S. The Audio Degradation Toolbox and its Application to Robustness Evaluation. In: Proceedings of the 14th International Society of Music Information Retrieval Conference (ISMIR 2013); 2013. p. 83–88.

[2] Fujishima T. Real Time Chord Recognition of Musical Sound: a System using Common Lisp Music. In: Proceedings of the International Computer Music Conference (ICMC 1999); 1999. p. 464–467.

[3] Mauch M, Dixon S. Simultaneous Estimation of Chords and Musical Context from Audio. IEEE Transactions on Audio, Speech, and Language Processing. 2010;18(6):1280–1289.

[4] Bartsch MA, Wakefield GH. Audio thumbnailing of popular music using chroma-based representations. IEEE Transactions on Multimedia. 2005;7(1):96–104.

[5] Müller M, Kurth F. Towards structural analysis of audio recordings in the presence of musical variations. EURASIP Journal on Applied Signal Processing. 2007;2007(1):163–163.

[6] Mauch M, Dixon S. Approximate note transcription for the improved identification of difficult chords. Proceedings of the 11th International Society for Music Information Retrieval Conference (ISMIR 2010). 2010;p. 135–140.

[7] Davis S, Mermelstein P. Comparison of parametric representations for monosyllabic word recognition in continuously spoken sentences. IEEE Transactions on Acoustics, Speech and Signal Processing. 1980;28(4):357–366.

[8] Foote JT. Content-Based Retrieval of Music and Audio. Proceedings of SPIE. 1997;138:138–147.

[9] Ito M, Donaldson RW. Zero-crossing measurements for analysis and recognition of speech sounds. IEEE Transactions on Audio and Electroacoustics. 1971;19(3):235–242.

[10] Gouyon F, Pachet F, Delerue O. On the use of zero-crossing rate for an application of classification of percussive sounds. In: Proceedings of the COST G-6 Conference on Digital Audio Effects (DAFX-00); 2000. p. 1–6.

[11] McLachlan GJ, Peel D. Finite Mixture Models. Hoboken, NJ: John Wiley & Sons; 2000.

[12] Burgoyne JA, Wild J, Fujinaga I. An expert ground-truth set for audio chord recognition and music analysis. In: Proceedings of the 12th International Conference on Music Information Retrieval (ISMIR 2011); 2011. p. 633–638.

[13] Papadopoulos H, Peeters G. Large-scale Study of Chord Estimation Algorithms Based on Chroma Representation and HMM. In: International Workshop on Content-Based Multimedia Indexing; 2007. p. 53–60.

[14] Mauch M, Dixon S, Harte C, Fields B, Casey M. Discovering chord idioms through Beatles and Real Book songs. In: Proceedings of the 8th International Conference on Music Information Retrieval (ISMIR 2007); 2007. p. 255–258.

[15] Blei DM, Ng AY, Jordan MI. Latent dirichlet allocation. Journal of Machine Learning Research. 2003;3:993–1022.

[16] Hornik K, Grün B. topicmodels: An R package for fitting topic models. Journal of Statistical Software. 2011;40(13):1–30.

[17] Rousseeuw PJ. Silhouettes: a graphical aid to the interpretation and validation of cluster analysis. Journal of computational and applied mathematics. 1987;20:53–65.

[18] Jost L. Entropy and diversity. Oikos. 2006;113(2):363–375.

[19] Erwin DH. DISPARITY: MORPHOLOGICAL PATTERN AND DEVELOPMENTAL CONTEXT. Palaeontology. 2007;50(1):57–73.

[20] Foote M. The evolution of morphological diversity. Annual Review of Ecology and Systematics. 1997;.

[21] Hallgrímsson B, Hall BK. Variation: A Central Concept in Biology. Academic Press; 2011.

[22] Foote J. Automatic audio segmentation using a measure of audio novelty. In: IEEE International Conference on Multimedia and Expo. vol. 1; 2000. p. 452–455.

[23] Smith JBL, Chew E. A Meta-Analysis of the MIREX Structure Segmentation Task. In: Proceedings of the 14th International Society for Music Information Retrieval Conference; 2013. .
